# Climate change and health in Southeast Asia – defining research priorities and the role of the Wellcome Trust Africa Asia Programmes

**DOI:** 10.12688/wellcomeopenres.17263.3

**Published:** 2022-09-05

**Authors:** Marc Choisy, Angela McBride, Mary Chambers, Chanh Ho Quang, Huy Nguyen Quang, Nguyen Thi Xuan Chau, Giang Nguyen Thi, Ana Bonell, Megan Evans, Damien Ming, Thanh Ngo-Duc, Pham Quang Thai, Duy Hoang Dang Giang, Ho Ngoc Dan Thanh, Hoang Ngoc Nhung, Rachel Lowe, Richard Maude, Iqbal Elyazar, Henry Surendra, Elizabeth A. Ashley, Louise Thwaites, H. Rogier van Doorn, Evelyne Kestelyn, Arjen M. Dondorp, Guy Thwaites, Nguyen Van Vinh Chau, Sophie Yacoub

**Affiliations:** 1 Oxford University Clinical Research Unit, Ho Chi Minh City and Hanoi, Vietnam; 2 Centre for Tropical Medicine and Global Health, University of Oxford, Oxford, UK; 3 Global Health and Infection, Brighton and Sussex Medical School, Brighton, UK; 4 Centre on Climate Change and Planetary Health, London School of Hygiene & Tropical Medicine, London, UK; 5 Centre for Environmental Health and Sustainability, University of Leicester, Leicester, UK; 6 Department of Infectious Disease, Imperial College London, London, UK; 7 University of Science and Technology of Hanoi, Vietnam Academy of Science and Technology, Hanoi, Vietnam; 8 National Institute of Hygiene and Epidemiology, Hanoi, Vietnam; 9 School of Preventative Medicine and Public Health, Hanoi Medical University, Hanoi, Vietnam; 10 Centre for Mathematical Modelling of Infectious Diseases, London School of Hygiene & Tropical Medicine, London, UK; 11 Barcelona Supercomputing Center, Barcelona, Spain; 12 Mahidol Oxford Tropical Medicine Research Unit, Bangkok, Thailand; 13 Eijkman-Oxford Clinical Research Unit, Jakarta, Indonesia; 14 Lao-Oxford-Mahosot Hospital-Wellcome Trust Research Unit, Vientiane, Lao People's Democratic Republic; 15 Hospital for Tropical Diseases, Ho Chi Minh CIty, Vietnam

**Keywords:** climate change, global warming, global heating, southeast asia, vietnam, thailand, laos, myanmar, indonesia

## Abstract

This article summarises a recent virtual meeting organised by the Oxford University Clinical Research Unit in Vietnam on the topic of climate change and health, bringing local partners, faculty and external collaborators together from across the Wellcome and Oxford networks. Attendees included invited local and global climate scientists, clinicians, modelers, epidemiologists and community engagement practitioners, with a view to setting priorities, identifying synergies and fostering collaborations to help define the regional climate and health research agenda. In this summary paper, we outline the major themes and topics that were identified and what will be needed to take forward this research for the next decade. We aim to take a broad, collaborative approach to including climate science in our current portfolio where it touches on infectious diseases now, and more broadly in our future research directions. We will focus on strengthening our research portfolio on climate-sensitive diseases, and supplement this with high quality data obtained from internal studies and external collaborations, obtained by multiple methods, ranging from traditional epidemiology to innovative technology and artificial intelligence and community-led research. Through timely agenda setting and involvement of local stakeholders, we aim to help support and shape research into global heating and health in the region.

## Introduction

In November 2021, 120 world leaders attended the 26 ^th^ UN Climate Change Conference (COP26) in Glasgow (UK), alongside more than 40,000 other stakeholders. This 12-day summit was dubbed as the ‘world’s best last chance to get runaway climate change under control’ ^1^; 153 participant countries agreed new 2030 emissions targets, and agreed plans to protect vulnerable communities and natural habitats, mobilise finances to make these goals achievable, and work together under the terms of the Paris agreement.

As the global community united to tackle this crisis, the Wellcome Trust also defined its research priorities around climate change and health; these are 1) to improve understanding of the effects of climate change on human health and 2) define the interventions and policies that can respond to the climate crisis in a way that protects and improves human health ^2^.

### The Wellcome Trust Africa and Asia programmes

The Wellcome Trust Africa and Asia programmes (AAP) are embedded in the world’s regions most affected by global heating, and most exposed to the impact of further climate disruptions. Southeast Asia is one of the most vulnerable regions of the world, with over 600 million people at risk of the results of global heating in the form of extreme weather events, flooding, droughts and fires, resulting in changing disease patterns, forced migration, food and water insecurity and climate refugees.

The AAPs in Southeast Asia already ^[Bibr ref-3]^ have extensive research expertise on climate-sensitive infections including vector-borne diseases like malaria and dengue, as well as diarrheal diseases, respiratory and novel emerging infections. A further strength lies in the long-standing academic relationships in the region and being embedded within local hospitals and public health institutions in Southeast Asia; these links render the AAPs well positioned to help set the regional research agenda to ensure that we and our partners are equipped to measure, respond to, and eventually develop mitigation strategies to minimise the impact of climate change on the health of the local population over the coming decades.

The AAPs have public engagement and policy teams with strong partnerships with communities, and well-established engagement platforms– for example networks to engage with school children and youth and long-standing community advisory boards, which will enable us to partner with the public and local communities to develop a grounded and locally relevant research agenda. 

### The meeting (7 ^th^ April 2021)

To complement global efforts through COP26, and in parallel with the Wellcome Trust defining their climate change and health agenda, a virtual meeting was organised by the Oxford University Clinical Research Unit (OUCRU) in Vietnam, which aimed to set priorities for the regional research agenda on climate and health, identify synergies in pre-existing work and future research interests, and foster collaborations between the faculty of the Wellcome and Oxford Networks and local partners. Attendees included 59 invited local and global climate scientists, clinicians, modelers, epidemiologists and community engagement practitioners. In this summary paper, we outline the major themes that were identified and what will be needed to take forward this research for the next decade. All attendees, whether attending in person or online, were welcomed to share opinions, ask and answer questions, or offer comments during the meeting. 

The meeting began with overviews of pre-existing research which has relevance to climate change and health presented by each of the units in the Asian AAP. This is summarised in the section ‘Climate and infectious disease research in the Asian Oxford Tropical network’. Once an inventory of research experience and opportunities had been achieved, the discussion moved on to setting the direction and agenda for future research. This is summarised in the section ‘Future directions and suggested research agenda for the Asian AAPs’.

## Meeting outcomes

### 1) Summarizing existing climate and infectious disease research in the Asian Oxford Tropical network

Over the last four decades, the Oxford Tropical Network in Southeast Asia has developed large research programmes in Vietnam, Thailand, Nepal, Indonesia, Cambodia, Myanmar and Laos, which have included a diverse portfolio of work on climate-sensitive diseases such as dengue, malaria, rickettsioses, diarrheic illnesses, respiratory and emerging infections ^4– 7^. The research has spanned both hospital-based and community studies (including long-term cohort studies, sentinel and passive surveillance), using various approaches ranging from clinical, microbiology and molecular epidemiology work through to entomology and mosquito-viral dynamics as well as mathematical modelling, innovative monitoring technologies and use of artificial intelligence. Although, until now, the interaction between meteorological factors and infectious diseases has not been a major research focus for these programmes, a number of important findings have been made, which, combined with decades of detailed datasets allows for more in-depth research on the interaction between climate and infectious diseases, to understand and predict changing disease burdens in the future.

Researchers at OUCRU-Vietnam are ideally placed to investigate the relationship between climate and infectious diseases, as Vietnam has a geographical range spanning almost the entire inter-tropical region (8° to 21° latitude) and an elevation ranging from sea level on the coast to more than 3,000 m in the central and northern highlands and hosts a high diversity of ecosystems. This, combined with a large population of almost 100 million (populating all the ecological regions equally), as well as a high intensity of infectious diseases in circulation (particularly influenza, dengue and diarrhoeal diseases), has enabled detailed modelling studies of these pathogens ^4, 8, 9^.

Researchers at Eijkman-Oxford Clinical Research Unit (EOCRU), Indonesian universities and international partners are investigating the association between agricultural land-use drivers and malaria. Changing patterns of human land use, particularly related to agricultural expansion and deforestation, are suggested to be the primary drivers behind the recent spread of *Plasmodium knowlesi* in humans ^10^. Strengthened practices for zoonotic malaria surveillance will assist in providing a local evidence base for policy makers to reduce disease transmission, facilitate sustainable agricultural development and enhance research capacity related to One Health methodologies.

With smaller units in Cambodia, Lao PDR, Myanmar and northern Thailand and study sites across the region, Mahidol Oxford Tropical Medicine Research Unit (MORU) in Bangkok, Thailand has led research on locally prevalent infectious diseases including malaria, melioidosis and scrub typhus over more than 4 decades. This has included working with national disease control programmes on analysis of routine malaria and dengue surveillance data to study the relationship between climate and disease patterns. Ongoing exploration of data from climate stations and satellite remote sensing together with case data at different spatial and temporal scales has revealed complex relationships that vary between locations. Data quality of climate, geographic and disease data are key determinants of the strengths of, and confidence in, these relationships. This learning is feeding into thinking about development of future analyses and predictive models of disease outbreaks and trends to help inform policy decisions.

This existing experience in investigating climate sensitive diseases, albeit not in dedicated climate studies, with the research infrastructure, and understanding of infectious disease dynamics and pathogenesis, sets the AAPs up well to further develop this into a firm and dedicated agenda for climate change studies. Their presence embedded in local health systems and experience so far shows the importance of long-term studies, the combination of data of multiple nature (entomology, epidemiology, environment, anthropology) and integrative analyses involving modelling, experiments and observational studies, all this at various spatial and temporal scales. In order to forge a dedicated research agenda for climate change within the Asia Network, the major climate-sensitive diseases currently investigated within the network (dengue, malaria, rickettsial infections, influenza and infectious diarrhoea) were discussed and areas were outlined upon which to build future research. We will also investigate whether other climate-sensitive infections currently not investigated by the network may should be added to our research agenda.

## Vector-borne diseases

### Dengue

The most notable example of the effect of meteorological conditions on infectious diseases concerns vector-borne infections such as dengue and malaria, which have been a major focus of research at OUCRU and MORU/EOCRU, respectively, over the last 20 years ^11^. Temperature, rainfall, humidity and changing land use affect the habitat availability of many arthropods, as well as various developmental stages of their life cycle. This has allowed us to understand and predict both the geographical range and the seasonality of many vector-borne diseases ^12^. In the past two decades several models have been developed for these two purposes. The most sophisticated ones explicitly account for the effect of climate change on the vector population dynamics, integrating data from entomological surveys ^13^. Researchers in OUCRU-Vietnam have assembled one of the largest dengue syndromic databases in the world (monthly dengue cases in 273 provinces of 8 countries of southeast Asia over 18 years), and were able to define in great detail how high temperature drives the spatial hierarchy in dengue epidemics across the region ^8^.

In addition to these modelling studies, OUCRU has a large translational programme of dengue research including clinical trials, innovative monitoring systems and pathogenesis studies, as well as investigations into the susceptibility of mosquitoes to dengue virus under various environmental conditions and manipulated treatment. The capacity of commensal intracellular *Wolbachia* bacteria to block dengue virus infection of mosquitoes has been a major theme at OUCRU, with studies comparing multiple *Wolbachia* strains, evolution of dengue virus in the presence of *Wolbachia*, and assessing *Wolbachia* to block vertical transmission of dengue ^14^.

With encouraging results from a randomised controlled trial in Indonesia, the world mosquito programme is now investigating the feasibility to mitigate the transmission of dengue virus using large scale release of *Wolbachia*-infected *Aedes aegypti* mosquitoes in Vietnam, Indonesia and Brazil ^15^. *Wolbachia* strains are sensitive to temperature, so the sustainability of this approach in a warming world will need to be continually evaluated. In that context, we are conducting experiments to assess the robustness of the *Wolbachia* effect under various temperatures and fluctuation regimens.

Recently, MORU Epidemiology Department has been working to develop models of the association between climate and dengue in Thailand and Myanmar working with the national disease control programmes using data from government climate stations and satellite remote sensing. Aiming to improve predicted trends in dengue incidence over time, including locations and timing of dengue outbreaks, these models incorporate a range of factors including reporting lags ^16^ and human mobility ^17^. Increasingly, MORU is also collecting diagnostic and demographic data on a wide range of infectious diseases, in projects across hundreds of villages in rural areas, for example through the South and Southeast Asian Community-based Trials Network (SEACTN) ^18^). These data can improve estimates of true disease incidence and distribution which will help to improve the predictive models.

### Malaria

Malaria has been a major research theme for MORU, with ongoing work on clinical trials, elimination interventions, pathogenesis trials, molecular biology, pharmacology, as well as epidemiology and mathematical modelling. MORU has a large and growing network of malaria study sites across Asia and Africa which provides access to a large quantity of high-quality clinical data. It also provides health services for malaria in several countries including in Myanmar by Medical Action Myanmar and Shoklo Malaria Research Unit and clinics in Thailand through the Borderland Health Foundation. These permit largescale collection of high-quality surveillance data to study patterns and trends and assess the impact of population level interventions. Data from Kayin State in Myanmar have been used to develop predictive models of malaria using climate data from satellite remote sensing. MORU has been working to support national malaria control programmes (NMCP) across the region. Under the Enhanced modelling for NMCP Decision-making in the Greater Mekong Subregion to Accelerate Malaria Elimination (ENDGAME) project these include development of models to answer specific policy questions ^19^. With a range of climate classes and seasonality across the region and variety of mosquito vector species, these relationships are complex and there is enormous scope for future research towards optimally incorporating current and future climate trends into predictive models of malaria trends to better predict timelines to elimination and identify locations at high risk of outbreaks.

Researchers at EOCRU along with their local collaborators have extensive experience in the zoonotic malaria parasite *P. knowlesi* and understanding its geographical distribution in terms of disease risk by human, animal and vector interaction ^10^. *P. knowlesi* is transmitted among macaques in a sylvatic cycle and zoonotically to humans by anopheline mosquitoes. EOCRU uses case data, climate variables, and land cover categories to generate fine-scale distribution maps for the three macaque host species ( *Macaca fascicularis*, *nemestrina* and *leonina*) and two mosquito vector complexes (Dirus and Leucosphyrus Complex). Conversion of intact forest into disturbed forest or the creation of vegetation mosaics, increases the probability that Leucosphyrus Complex will thrive at these locations, as well as bringing humans into these areas.

### Rickettsial infections

Rickettsial infections are widely distributed globally and transmitted by ticks, mites, lice, and fleas. Climate change may lead to changes in the environment which affect host and vector abundance and as a result impact on disease incidence. In a longitudinal study (2003 to 2017) from the Lao PDR, trends of murine and scrub typhus incidence were associated with temperature and rainfall respectively, suggesting that global heating and increased precipitation may expand the distribution and burden of these diseases ^20^.

## Other climate-sensitive diseases

### Influenza

In addition to vector-borne and environmental infectious diseases, meteorological conditions can also affect the transmission of human-to-human transmitted respiratory infectious diseases such as measles ^21^ or influenza ^22, 23^), either by affecting the survival of the virus in the air, or by changing the behaviour of the human host. Controlled experiments coupled with epidemiological modelling allow to decipher these effects. A model developed and calibrated at OUCRU-Vietnam showed that absolute humidity is the main driver of the intensity of seasonal variation of influenza-like illness (ILI) ^9^. The model calibrated on Vietnamese data successfully predicted the epidemiological regimen of influenza in over 75 locations around the world. This was possible because of the large diversity of climatic conditions in Vietnam, representing a substantial proportion of the diversity of climates.

### Infectious diarrhoea

Another class of infectious diseases that have been shown to be influenced by meteorological variables are those with (partial) environmental transmission. *Vibrio cholerae* is a bacterium that lives on the surface of copepods in estuaries. When such bacteria are ingested by humans, they cause cholera. This can then initiate human-to-human orofecal transmission chains and outbreaks among human populations, even in locations remote from the coast. An increase in estuary / sea surface temperature can trigger a demographic explosion of copepod populations on the surface on which *V. cholerae* thrive, thereby dramatically increasing the probability of human ingestion and triggering an outbreak. The risk of cholera outbreaks have been successfully predicted from monitoring sea surface temperature that can easily be achieved in real-time from satellite measurements ^24^.

A study combining epidemiology, hydrology, microbiology, and anthropology to investigate the seasonal epidemiology of diarrheal infections in northern Laos found that enteric bacteria concentration in river water was higher during flooding. However, paradoxically, the incidence of diarrhea was higher in the dry season than the rainy season, likely driven by the population forced to use highly contaminated surface water instead of clean naturally filtered well water, highlighting the importance of community and behavioural studies and engagement ^4^.

## Challenges of climate change models and infections

Disease models are increasingly used to tackle the more challenging task of anticipating the consequences of climate change on the burden of infectious diseases ^25, 26^. The challenge comes from the fact that we are dealing with an extrapolation exercise for conditions that have never been experienced before and thus have no data available yet. Mathematical models explicitly accounting for the biological mechanisms of each life-cycle step are expected to produce more robust predictions than classical black-boxes approaches, such as classical statistical analysis or even more sophisticated machine learning approaches ^27^. Difficulties when exploring the link between meteorological conditions and infectious diseases are that transmission also depends on a myriad of human factors such as population density, susceptibility, age structure and mobility etc., which makes the identification of the exact effects of meteorological variables more complicated ^28, 29^.

Furthermore, the effects of meteorological conditions are often non-linear ^30, 31^. In general, biological processes have an optimal range rather than a linear relationship with temperature ^32^. Effects can also be multiple, partially contradictory, with various delays, and also more or less direct. Heavy rainfalls may for example wash-up mosquito populations in the short term but prepare for optimal breeding sites and humidity conditions in the longer term ^33^. As for assessing the epidemiological impact of climate change, an additional complication relates to the fact that the host, the pathogen and the vector can adapt to these changes, and this has already been documented ^34^. Such adaptations clearly add a large degree of uncertainty to any prediction that can be made. So far, we have discussed the effect of meteorological variables on the vector only, but it is also well documented that the development time of the etiologic agent in the vector (extrinsic period) is highly dependent on temperature too ^35^.

## 2) Future directions and suggested research agenda for the Asian AAPs

**1) The need for high quality long-term longitudinal data**

Climate change and its effects occur over multiple years and decades, and our previous research has shown the necessity of data collected over long periods of time to be able to conduct attribution studies. Furthermore, to support accurate understanding of the impact of global heating, it is important that the data used are accurate at multiple spatial scales (global, regional, local). We discussed several different mechanisms of obtaining data to complement clinical and laboratory studies; these include data collection systems based on satellite technology incorporating meteorological, topological, ecological data, downscaling data from global models to produce local, high-resolution data and using mHealth/drones for data gathering at the local level.

**Figure 1.**
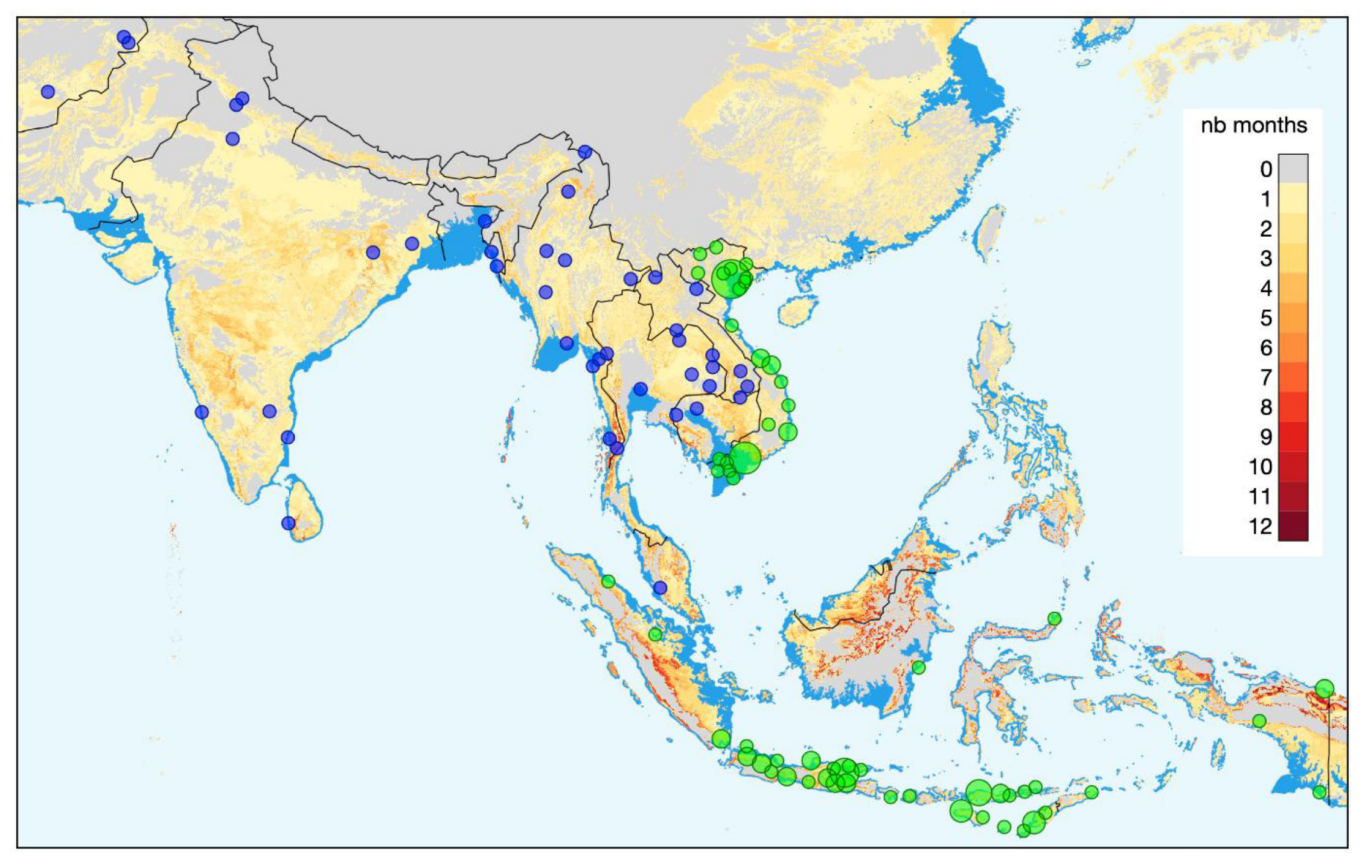
Locations of the Oxford University Clinical Research Unit (green) and Mahidol Oxford Tropical Medicine Research Unit (MORU) (blue) study sites. MORU additionally has study sites in Eastern Africa that are not shown here. The background colours of the map represent the additional number of months per year that are predicted to have a monthly average maximum temperature above 30°C over the 2081–2100 time period (based on data simulated by the CNRM-CM6-1 GCM model with the SSP 126 and obtained from WorldClim. The dark blue color shows the coastal area lost to sea by 2100.

A crucial part of recognizing the potential health impacts of climate related hazards is identifying changes in disease patterns over time and linking these to climate related parameters, land-use change/ecology changes/biodiversity loss. This then allows development of accurate models to predict future health threats, and can result in health system and policy changes to prepare for these. However, for this to be possible, data collection with sufficient geographical density at different levels of the health system is required. The Wellcome AAPs in Asia are well positioned to capture such data in their wide network of intensive care units (ICUs), hospitals, and community-based health workers. Wellcome Innovations supports a large Asian ICU Flagship programme, jointly led by OUCRU and MORU. Part of the programme has been the establishment of an electronic ICU registry, which captures information on diagnosis and disease severity. The registry is currently running in 15 Asian (as well as 9 African) countries, including 231 hospitals, and still expanding. A parallel Wellcome Innovations Flagship programme supports the Southeast Asia Community-Based Trials Network ^36^, which comprises large networks of community health workers in Myanmar, Laos, and Cambodia. In Vietnam and Indonesia, networks encompass both human and animal health monitoring at community level. In addition, OUCRU and MORU research units together have an extensive network of over 100 clinical trial sites across 11 Asian countries ( Figure 1).

With this infrastructure in place, we plan for the next five years to systematically capture the changes in disease burden and disease patterns, as well as climate related data. This will deliver an invaluable data source for interrogation with the aim to identify specific climate hazard related health impacts. This will then serve as a starting point to design interventions to counter these adverse health outcomes.

Another challenge regarding investigation of the climate effects on health in general is that climatic and health data are often produced at different spatial and temporal scales and resolutions ^37^. In order to address this issue, we will explore the combination of (i) mechanistic models of the effect of climatic variables on the biological processes of these epidemiological systems and (ii) more agnostic machine-learning-type of modelling in order to identify relevant proxies across scales. The general approach is inspired from methodologies currently developed in downscaling research.

**2) The need for local and international interdisciplinary collaboration**

To supplement high quality clinical and epidemiological data obtained by traditional research methods, cross-disciplinary collaboration will be required to mount the broad approach for a such a challenge such as climate change. Internal collaboration between clinicians, data scientists and mathematical modelling teams should be supplemented by cross discipline work with anthropology, microbiology, entomology and public health. Collaboration between the Wellcome AAPs in both continents will help to optimise use of resources, align data collection and linkage across diverse datasets to provide a more regionally holistic picture.

In addition, obtaining and processing high quality meteorological and climate data will require collaboration outside of our established network. We have identified several projects underway by local and international research groups using advanced mapping technologies, early warning systems and artificial intelligence to model accurate impacts of climate change on both local, regional and national level. For example, a dengue warning system from London School of Hygiene and Tropical Medicine (LSHTM) ^38^, and the Vietnam space mapping technology. Working with meteorologists, hydrologists, environmental scientists, engineers and anthropologists may also be key to implementing sustainable interventions.

**3) The need for innovation - mhealth**

Climate related data are collected through a variety of methods, including weather stations and satellites. Recent technological innovations offer novel and improved approaches to both the surveillance and response to climate-driven health challenges. Mobile Health (often abbreviated to mHealth) incorporates the use of mobile devices, patient monitoring devices, and other wireless technologies. Use of such devices enable a range of functions, such as sending and receiving messages, as well as more advanced technologies, including Global Positioning Systems (GPS). Such devices and technologies enable more timely and accurate collection of data, particularly in remote regions, and offer many potential applications with regards to health promotion and protection. Satellite systems monitor the meteorological, environmental, and physiological status that may influence the transmission of certain infectious diseases. This data can be used to augment meteorological data collected at regional or national level and enable the development of effective surveillance and early warning systems. When combined with pattern recognition and disease trends, satellite data may enable disease outbreaks to be predicted in advance ^39^.

We plan to integrate these unique approaches to both the surveillance and response to climate-driven health challenges, incorporating novel digital technologies, remote patient monitors, mHealth initiatives, artificial intelligence (AI) methodologies with satellite-assisted data systems. For example, we intend to integrate AI algorithms into existing surveillance systems, which will enable faster and more accurate processing of large amounts of data, resulting in more precise detection and prediction of disease outbreaks.

**4) Need for individual as well as population level research**

Changes in weather patterns and increasing frequency of extreme weather events exert important, diverse effects at an individual patient level. Increased hospitalisations and mortality are directly attributable to human-induced global heating ^40, 41^. In regions experiencing higher ambient temperatures (i.e. temperatures that people are experiencing, as opposed to temperature from weather stations that are measured in very specific controlled conditions), heat stress causes many health impacts including deranged physiological processes such as water handling and electrolyte balance. Direct heat stresses also affect cardiovascular ^42^ and renal health ^43^, and exacerbate dehydration and shock in infections. Southeast Asia and other tropical regions are already experiencing the health impacts of extreme heat, but are predicted to be more severely affected in coming years ^40, 44^. Estimations of the impact of occupational heat (e.g., in outdoor workers) in South East Asia by 2050 are extreme ^45, 46^. Research from the military and sports medicine indicate that wearable technology and ingestible core telemetry pills can be used to identify heat strain early, allowing mitigation strategies such as active cooling ^47^. A priority area for research going forward is identifying at-risk groups like outdoor workers, pregnant women, children and the elderly and tailoring solutions to individual patients ^48^.

For hospitalised patients, clinical decision-making is affected by climate variables. Established relationships between seasonality and prevalence of infections are implicit knowledge taken into account by clinicians: for example, in wet season the increased prevalence of dengue in SE Asia has implications on the diagnosis and management of acute febrile illnesses and affects effectiveness of treatment and diagnostics as a function of positive and negative predictive value. Changing climatic variables can present challenges for vector-borne diseases by establishing new areas of autochthonous transmission, as well as changing seasonal epidemiology. The development of data-driven tools such as clinical decision support systems (CDSS) aimed at providing support in the management of illness will increasingly need to explicitly acknowledge and account for these variables, and integrate with outbreak models. Thus, we envisage that CDSS of the future would be tailored and contextualised to geographic location, season and climate.

**5) The need for public and policy engagement**

The final area identified as a key component of the future AAP programme is that of public and policy engagement, with the goals of (1) better understanding the potential impact of climate change on wellbeing and mental health, (2) learning the research priorities of vulnerable communities in relation to climate change, and (3) expanding our collaborations outside of the scientific community (4) Engaging with government and key policy-makers.

Although climate change is recognised as a health emergency, this is predominantly referred to as a physical health emergency, often with little reference to mental health. However, there is increasing evidence that both the drivers and consequences of climate change can threaten emotional wellbeing ^49^. In addition, eco-anxiety (worry about the environment) can cause psychological distress. Eco-anxiety, defined by the American Psychological Association as “a chronic fear of environmental doom”, is not recognised as a disorder and in fact, may be an appropriate reaction to the climate emergency. Indeed, when linked to increased activism and a sense of empowerment, it can have positive impacts on mental health ^50^. Research on the psychological effects in young people is scarce although they are likely to be disproportionately affected, and the few published studies have focused on youth in the Global North or amongst indigenous peoples ^51– 53^. However, an increase in online discussions and posts on this topic from Asian and African young people suggest that despite the lack of data, this anxiety isn’t a uniquely first world problem and is likely to be linked to the rising cases of poor mental health in children and youth in low and middle income countries ^54– 56^.

Engagement activities will need to be tailored to sociocultural settings across the networks – and therefore need to be designed and driven in collaboration with the community. We plan to engage with the public, particularly youth, who are major stakeholders in climate change and empower them to take action, advocate for change and develop solutions. Climate change and mental health are priority areas already identified by the OUCRU facilitated Youth Engagement with Science (YES) group – a collective of over 800 Vietnamese young people.

In addition to community engagement, we also have an active policy engagement team within the AAPs. In Vietnam we are currently working with various government institutions including; the ministry of Health (MoH), department of health (DoH) provincial CDCs, national, provincial and district hospitals, national public health agencies (Pasteur Institute, NIHE in Hanoi), and the National Bureau of Meteorology and of Natural Resources and Environment (MONRE and DONRE). Going forward we plan to increase engagement and discussion around our climate and health research with these key stake-holders and policy-makers at national and provincial levels

Finally, we do not wish to limit our collaboration to the scientific community. Through collaboration with other stakeholders such as non-governmental organisations, community-based organisations and embassies, we aim to expand our reach and encourage action to be taken in local and national policy-making spheres.

## Summary

Going forward, we aim to take a broad, collaborative approach to including climate science in our current portfolio where it touches on infectious diseases now, and more broadly in our future research directions. Box 1 summarises our next steps and research priorities. We will focus on building our research upon climate-sensitive diseases with which we already have considerable experience, and supplement this with high quality data obtained from internal studies and external collaborations, obtained by multiple methods, ranging from traditional epidemiology to innovative technology and artificial intelligence and community-led research. Through timely agenda setting and involvement of local stakeholders, we aim to help support and shape research into global heating and health in the region.

**Box 1. Next steps and priorities**

   •   To build a regional network of climate and health collaborators/stakeholders

   •   Identify local and regional climate-sensitive infectious disease risk, with the eventual aim of enabling the design and testing of interventions.

   •   Develop innovative solutions to climate-driven health challenges, through integration of novel digital technologies, remote patient monitors, mHealth initiatives and artificial intelligence (AI) methodologies, including:

   -   AI-assisted surveillance /satellite-assisted data systems for geospatial risk mapping of climate-sensitive diseases (including vector-borne and water-borne diseases)

   -   Incorporate meteorological data into clinical decision support systems for individual management of acute febrile illnesses.

   •   Develop engagement activities with vulnerable communities to understand their priorities and the potential impact of climate change on mental health, facilitated by the OUCRU Youth Engagement with Science programme.

   •   Expand our communication and engagement with government and non-governmental organisations, community-based organisations and embassies to develop action to be taken in local and national policy-making spheres.

## Data availability

No data are associated with this article.
