# Supplementary material for: Climate change and health in Southeast Asia – defining research priorities and the role of the Wellcome Trust Africa Asia Programmes
Source: Wellcome Open Res. 2022 Sep 5;6:278. Originally published 2021 Oct 18. [Version 3] doi: 10.12688/wellcomeopenres.17263.3 (PMC9493397; doi:10.12688/wellcomeopenres.17263.3)
Supplement: Peer review history

# Peer review: Reviewer response for version 3

**DOI:** 10.21956/wellcomeopenres.20348.r52243

**Published:** 2022-09-21

**Authors:** Hannah Nissan

**Affiliations:** 1 Grantham Institute for Climate Change and the Environment, London School of Economics and Political Science, London, UK; 2 Climate and Resilience Hub, Willis Towers Watson, London, UK

No further comments.

Does the article adequately reference differing views and opinions?

Partly

Are all factual statements correct, and are statements and arguments made adequately supported by citations?

Yes

Is the Open Letter written in accessible language?

Yes

Where applicable, are recommendations and next steps explained clearly for others to follow?

Partly

Is the rationale for the Open Letter provided in sufficient detail?

No

Reviewer Expertise:

Climate science and modelling; climate information and its use for public health control programmes; climate change.

I confirm that I have read this submission and believe that I have an appropriate level of expertise to confirm that it is of an acceptable scientific standard.

# Peer review: Reviewer response for version 1

**DOI:** 10.21956/wellcomeopenres.19080.r51313

**Published:** 2022-07-04

**Authors:** Paul Lester Chua

**Affiliations:** 1 Department of Global Health Policy, Graduate School of Medicine, The University of Tokyo, Tokyo, Japan

This open letter is a timely piece to disseminate and encourage efforts in conducting climate change and health research in Southeast Asia (SEA), wherein most countries are vulnerable to climate change impacts. The Asian Oxford Tropical network is indeed in a good position to perform this work with the good quality data they collected or can collect to the benefit of climate change and health researchers (e.g. modelers). One concern I have in this open letter is the message of “exclusivity” within the network they have (presumably driven by Western counterparts) rather than consider expanding collaborations with existing efforts from all nations in SEA (i.e. ASEAN countries). The authors did mention local collaboration but seem like they only refer to cross disciplines and community engagement. The proposed directions may leave out certain research efforts (e.g. existing local work or datasets) in and outside the SEA countries they work with. This message may be a personal observation and was never intended since this open letter solely reflects the discussions that came out from the participants of the virtual meeting. Nonetheless, the authors' pursuit of climate change and health research in SEA is still a commendable endeavour making this open letter relevant.

**Some specific comments:**

**Introduction**

- The first sentence in the first paragraph needs to be updated: " *At the end of 2021, more than 190 world leaders will attend the 26th UN Climate Change Conference (COP26) in Glasgow (UK), alongside thousands of other stakeholders".*
- In the first sentence of the second paragraph, does Wellcome Trust intend to carve its own rather than align to the global efforts? " *the Wellcome Trust is defining its own research priorities and agenda around climate change and health".*
- If possible, please include citations in the third and fourth paragraphs like a webpage summarising AAP work.
- It may be better to mention the date of the virtual meeting and the number of participants.

**Climate and infectious disease research in the Asian Oxford Tropical network**

- As mentioned in the last sentence of this section, how were major climate-sensitive diseases selected in SEA? Were they selected based on the perceptions or expertise of the participants? There is a possibility there are other “major” climate-sensitive diseases being missed out.
- In Figure 1, why use SSP 1-RCP 2.6? Would it be closer to reality using SSP2-RCP 4.5? Why also limit to temperature? Would climate categories or precipitation be created as well?

**Future directions and suggested research agenda for the Asian AAPs**

- Would the authors be confident to say that the AAP's wide network of intensive care units (ICUs), hospitals, and community-based health workers will be able to capture the major climate-sensitive infectious diseases? Are governmental health facilities excluded from this AAP work?
- In the last paragraph (and in the entirety of the piece), there was no mention of collaborating or interacting with governments. It is understandable that SEA governments are usually not open to research collaboration because of many reasons, but translating research into policy requires their cooperation. It may be good to consider how to interact with them in the future.

**Explanation on some of the set questions:**

- ***Does the article adequately reference differing views and opinions?***
  The point of view of the open letter only reflects the discussion in the virtual meeting and there are no opposing views/opinions.
- ***Are all factual statements correct, and are statements and arguments made adequately supported by citations?***
  The strong statements on the achievements of the AAP and/or Asian Oxford Network would be better to be fully referenced like in the third and fourth paragraphs of the “Introduction” and first, third, fourth, and fifth paragraphs of “Climate and infectious disease research in the Asian Oxford Tropical network”.

Does the article adequately reference differing views and opinions?

Partly

Are all factual statements correct, and are statements and arguments made adequately supported by citations?

Partly

Is the Open Letter written in accessible language?

Yes

Where applicable, are recommendations and next steps explained clearly for others to follow?

Yes

Is the rationale for the Open Letter provided in sufficient detail?

Yes

Reviewer Expertise:

Climate change and health

I confirm that I have read this submission and believe that I have an appropriate level of expertise to confirm that it is of an acceptable scientific standard.

# Response

**Published:** 2022-08-18

**Authors:** Angela McBride

**Affiliations:** Oxford University Clinical Research Unit, Vietnam

Many thanks for taking the time to review this open letter.

**This open letter is a timely piece to disseminate and encourage efforts in conducting climate change and health research in Southeast Asia (SEA), wherein most countries are vulnerable to climate change impacts. Thea Asian Oxford Tropical network is indeed in a good position to perform this work with the good quality data they collected or can collect to the benefit of climate change and health researchers (e.g. modelers). One concern I have in this open letter is the message of “exclusivity” within the network they have (presumably driven by Western counterparts) rather than consider expanding collaboration with existing efforts from all nations in SEA (i.e. ASEAN countries). The authors did mention local collaboration but seem like they only refer to cross disciplines and community engagement. The proposed directions may leave out certain research efforts (e.g. existing local work or datasets) in and outside the SEA countries they work with. This message may be a personal observation and was never intended since this open letter solely reflects the discussions that came out from the participants of the virtual meeting. Nonetheless, the authors’ pursuit of climate change and health research in SEA is still a commendable endeavour making this open letter relevant.**

 Author response: Thank you for your comment. Our network is by no means ‘exclusive’; we have extensive local collaborators within Vietnam and other ASEAN countries (as explained on p8). This initial meeting included our current local partners, but one of the outcomes of the meeting, (as stated in Box 1) and one of our first priorities is ‘to build a regional network of climate and health collaborators/stakeholders.’

**Some specific comments:**

**Introduction**

**The first sentence in the first paragraph needs to be updated: “At the end of 2021, more than 190 world leaders will attend the 26 ^th^ UN Climate Change Conference (COP26) in Glasgow (UK), alongside thousands of other stakeholders”.**

 Author response: Good point. Updated.

**In the first sentence of the second paragraph, does Wellcome Trust intend to carve its own rather than align to the global efforts? “the Wellcome Trust is defining its own research priorities and agenda around climate change and health”.**

 Author response: No, Wellcome certainly aligns to the global efforts. What we meant here is that Wellcome is developing a research agenda within that global effort and that this agenda is focused on the consequences of climate change on health. Hopefully, the text reflects this more clearly now.

**If possible, please include citations in the third and fourth paragraphs like a webpage summarizing AAP work.**

 Author response: A link has been provided to the Wellcome Trust overview of the Africa Asia Programmes.

**It may be better to mention the date of the virtual meeting and the number of participants.**

 Author response: This has been added.

**Climate and infectious disease research in the Asian Oxford Tropical network**

**As mentioned in the last sentence of this section, how were major climate-sensitive diseases selected in SEA? Were they selected based on the perceptions or expertise of the participants? There is a possibility there are other “major” climate-sensitive diseases being missed out.**

 Author response: These diseases were selected based on the diseases that AAP is currently working on and for which climate sensitivity has been shown by us or others. It is certainly not exhaustive and it is possible that some key infections are missed out; if this is the case, then it is also one of our goals to identify and potentially develop new research on them.

**In Figure 1, why use SSP 1-RCP 2.6? Would it be closer to reality using SSP2-RCP 4.5? Why also limit to temperature? Would climate categories or precipitation be created as well?**

 Author response: Good point. The new version of Figure 1 now shows both temperature change and rise in sea level which is expected to be of primary concern given that most of the population actually lives by the sea in this part of the world. As for the choice of the scenario, that could indeed be argued. SSP2-RCP 4.5 is indeed closer to reality unfortunately. We chose SSP1-RCP 2.6 for the figure in order to provide a conservative projection.

**Future directions and suggested research agenda for the Asian AAPs**

**Would the authors be confident to say that the AAP’s wide network of intensive care units (ICUs), hospitals and community based health workers will be able to capture the major climate-sensitive infectious diseases? Are governmental health facilities excluded from this AAP work?**

 Author response: No, government health facilities are not excluded, in fact, many of the AAPs are based within government hospitals (e.g. the Hospital for Tropical Diseases in Ho Chi Minh City) and our network covers the full range of healthcare settings from ICUs through to community health care centers. So we should be well placed to capture changing patterns of climate-sensitive diseases presenting to these healthcare facilities.

**In the last paragraph (and in the entirety of the piece), there was no mention of collaborating or interacting with governments. It is understandable that SEA governments are usually not open to research collaboration because of many reasons, but translating research into policy requires their cooperation. It may be good to consider how to interact with them in the future.**

 Author response: Thank you for highlighting this, as this is an extremely important area of work for the AAPs and we have a very active engagement and policy team. In Vietnam, we are currently working with various government institutions including; the Ministry and Department of Health (MoH and DoH), provincial CDCs, national, provincial, and district hospitals, national public health agencies (Pasteur Institute, NIHE in Hanoi), and the National Bureau of Meteorology and of Natural Resources and Environment (MONRE and DONRE). We have updated the manuscript on p15.

# Peer review: Reviewer response for version 1

**DOI:** 10.21956/wellcomeopenres.19080.r50668

**Published:** 2022-06-20

**Authors:** Hannah Nissan

**Affiliations:** 1 Grantham Institute for Climate Change and the Environment, London School of Economics and Political Science, London, UK; 2 Climate and Resilience Hub, Willis Towers Watson, London, UK

**Detailed explanation for the answers to the set questions**

**Is the rationale for the Open Letter provided in sufficient detail?**

 The article could be improved by providing more comprehensive background information about the rationale for the meeting it describes and what it achieved (see below).

**Does the article adequately reference differing views and opinions?**

 The article does not cite differences of opinion; perhaps none were raised in the meeting. I am not sure exactly of the makeup of the team involved, but it appears that some perspectives may be absent – e.g. climate and meteorological expertise/services, and operational disease control programmes. It would be advisable to engage these communities in a genuine dialogue before finalising a research agenda and way forward.

**Are all factual statements correct, and are statements and arguments made adequately supported by citations?**

 To the best of my knowledge, yes, except for a few statements which require citations (see below).

**Is the Open Letter written in accessible language?**

 Yes. In general, the paper would benefit from more precise language in a few places.

**Where applicable, are recommendations and next steps explained clearly for others to follow?**

 Recommendations are clearly included, but these should be more precisely described, and an explanation given for how they will be achieved.

**Report**

***Introduction***

 In addition to improving the overall framing of the paper and providing more detail about the rationale for the meeting (see Q1 above), the paper would benefit from a description of the process that was followed to engage participants before and during the meeting to elicit responses? How were differing viewpoints reconciled? The article does not express any differences of opinion. Was everyone in agreement?

 A point about structure: it is not currently clear where the description of the outcomes of the meeting begin (until one arrives at the section “Future directions and suggested research agenda for the Asian AAPs” on page 6). The section 'climate and infectious disease research in the Asian Oxford Tropical Network' could be describing the outcomes of the meeting or part of the background literature. Some clarity over the different sections of the article would make it easier for the reader. I would also suggest including an overview of the article structure in the introduction for further clarity.

 Could the relationship between the AAPs and the Oxford Tropical Network be clarified?

***Climate and infectious disease research in the Asian Oxford Tropical network*******

 A reference is needed for the statement: “Changing patterns of human land use, particularly related to agricultural expansion and deforestation, are suggested to be the primary drivers behind the recent spread of *Plasmodium knowlesi *in humans.”

 First paragraph on page 5: “Data quality of climate, geographic and disease data are key determinants of the strengths of these relationships.” Wouldn’t data quality be a key determinant of confidence in an assessed relationship, not just its strength?

 Some claims in the second paragraph of page 5 require substantiation: “Their presence embedded in local health systems and experience so far shows the importance of long-term studies, the combination of data of multiple nature (entomology, epidemiology, environment, anthropology) and integrative analyses involving modelling, experiments and observational studies, all this at various spatial and temporal scales.” Where do these findings come from?

***Vector-borne diseases***

 The structure is not clear. This section reads as a summary of existing work on these topics rather than a research agenda. The reader expects a research agenda, given the framing in the abstract and introduction. Please clarify where the background literature review ends and the research agenda begins.

***Challenges of climate change models and infections***

 This section provides a thorough discussion of the challenges from the perspective of infectious disease modelling, but there are major challenges around the uncertainties arising from the boundary conditions that are not discussed (i.e. the climate forcings used for these types of modelling studies). Appropriate expertise within the research team is essential adequately to account for the complexities of climate modelling and its uncertainties. I cannot tell for sure, but the absence of a discussion of the strengths and limitations of climate models implies that this expertise is not represented in the author team. This imbalance should be addressed before finalising any research agenda, in particular before deciding on experimental designs and analytical approaches.

***Future directions and suggested research agenda for the Asian AAPs***

**1)**** **The need for high-quality, long-term, longitudinal data**

 The authors discuss the need for data at multiple spatial scales and densities. Another major challenge with using longitudinal population data for climate-health research is that the two datasets are often sampled at inappropriate temporal frequencies to capture the relationships between climate/weather drivers and health outcomes. Did the participants discuss these challenges and how to overcome them to achieve their research agenda? They may wish to consult a recent report produced for the Wellcome Trust on this topic ^1^.

**2) The need for local and international interdisciplinary collaboration**

 Has the author team considered including the relevant National Meteorological Services, not just researchers? This would be advisable.

**4) Need for individual as well as population level research**

 “In regions experiencing higher ambient temperatures, heat stress causes many health impacts including deranged physiological processes, such as water handling and electrolyte balance.” What is meant by 'ambient' in this sentence? Deaths occur from the heat at relatively moderate temperatures, not only extremes.

**5) The need for community engagement**

 The participants identified three goals of community engagement. Supporting operational disease control programs was not mentioned. Did the participants discuss this? Supporting operational disease programming would surely represent an important perspective when setting the research agenda if the intention is to reduce climate-related disease burdens (see also comments below).

**Box: Next steps and priorities**

 This box is a useful addition to the article but would benefit from being specified more precisely. How will these be achieved? The second point, in particular, is very far-reaching. It seems that you have already prioritised climate-sensitive diseases in this paper, and by the topics that you chose to discuss during the meeting. Is this point more about better understanding and quantifying the risks from these diseases?

 This is the first place where interventions are mentioned. If the ultimate aim is to reduce climate-related disease risks, surely supporting practical disease programming and operations should be important to the research agenda. It is of course necessary to understand climate-health associations via traditional epidemiological research, but also to understand both where the entry points are for designing new interventions and (often overlooked) how climate could interfere with existing disease control and elimination efforts. Reducing these risks will involve some use of climate data and potentially forecasts, and research questions can be designed with these interventions in mind. Failing to include them at this stage would miss an important opportunity to ensure that the research outcomes are useful for developing effective interventions.

 One further question/clarification: why are youth groups specifically the focus of the engagement about climate and mental health?

Does the article adequately reference differing views and opinions?

Partly

Are all factual statements correct, and are statements and arguments made adequately supported by citations?

Yes

Is the Open Letter written in accessible language?

Yes

Where applicable, are recommendations and next steps explained clearly for others to follow?

Partly

Is the rationale for the Open Letter provided in sufficient detail?

No

Reviewer Expertise:

Climate science and modelling; climate information and its use for public health control programmes; climate change.

I confirm that I have read this submission and believe that I have an appropriate level of expertise to confirm that it is of an acceptable scientific standard, however I have significant reservations, as outlined above.

## References

[1] : Combining climate and health data: challenges and opportunities for longitudinal population studies. *Wellcome Trust* .2022; Reference source

# Response

**Published:** 2022-08-18

**Authors:** Angela McBride

**Affiliations:** Oxford University Clinical Research Unit, Vietnam

Many thanks for taking the time to review this open letter. 

**In addition to improving the overall framing of the paper and providing more details about the rationale for the meeting (see Q1 above), the paper would benefit from a description of the process that was followed to engage participants before and during the meeting to elicit responses? How were differing viewpoints reconciled? The article does not express any differences of opinion. Was everyone in agreement?**

 Author response: A broad range of opinions were aired at the meeting, mostly relating to the variety of participant backgrounds (infectious disease doctors, mathematical modellers, climate scientists, etc.), but the overarching goals certainly appeared to be aligned, and complementary rather than contradictory. 

 We will continue to make sure that a broad range of perspectives and expertise is represented within our collaborative projects. For example, within Vietnam, we will be working with the Vietnam Institute of Meteorology, Hydrology and Climate Change (IMHEN), the National Institute of Hygiene and Epidemiology (NIHE), the Pasteur Institute of Ho Chi Minh City, and the Centers of Disease Control at provincial levels (in particular, Hanoi and Ho Chi Minh City). 

**A point about structure: it is not currently clear where the description of the outcomes of the meeting begin (until one arrives at the section “Future directions and suggested research agenda for the Asian AAPs” on page 6). The section ‘climate and infectious disease research in the Asian Oxford Tropical Network’ could be describing the outcomes of the meeting or part of the background literature. Some clarity over the different sections of the article would make it easier for the reader. I would also suggest including an overview of the article structure in the introduction for further clarity.**

 Author response: Regarding clarity of structure, the revised article introduction includes an overview as suggested. To clarify, the Asian AAP is a very broad programme (more than 1000 staff) spread over six countries in Southeast Asia. This is clearly an asset for us, as highlighted in the article. But it also represents a challenge to maintain cohesion in research programmes across the AAP. Since the intersection between climate change and infectious diseases is a new research topic for the Asian AAP, the first part of the meeting actually focused on making an inventory of opportunities for the Asian AAP to work on the topic – this is what is transcribed in the article until the section “Future directions and suggested research agenda for the Asian AAPs”.

**Could the relationship between the AAPs and the Oxford Tropical Network be clarified?**

 Author response: AAP is a funding programme of The Wellcome Trust, whereas the Oxford Tropical Network (OTN) is a research network of the University of Oxford. A substantial part of the research done in the OTN is funded by the AAP, but the AAP also funds research carried out by other institutions outside of the University of Oxford (e.g. LSHTM, University of Liverpool).

**A reference is needed for the statement “Changing patterns of human land use, particularly related to agricultural expansion and deforestation, are suggested to be the primary drivers behind the recent spread of Plasmodium knowlesi in humans”.**

 Author response: This is reference 17.

**First paragraph on page 5: “Data quality of climate, geographic and disease data are key determinants of the strengths of these relationships.”. Wouldn’t data quality be a key determinant of confidence in an assessed relationship, not just its strength?**

 Author response: Certainly, thanks for the comment. We will alter the text accordingly.

**Some claims in the second paragraph of page 5 require substantiation: “Their presence embedded in local health systems and experience so far shows the importance of long-term studies. The combination of data of multiple nature (entomology, epidemiology, environment, anthropology) and integrative analyses involving modelling, experiments and observational studies, all this at various spatial and temporal scales.” Where do these findings come from?**

 Author response: This comes from our past research experience in the region.

***Vector-borne diseases***

**The structure is not clear. This section reads as a summary of existing work on these topics rather than a research agenda. The reader expects a research agenda, given the framing in the abstract and introduction. Please clarify where the background literature review ends and the research agenda begins.**

 Author response: Yes, this is a summary of past and existing work in the Asian AAP. This is the case not only for this paragraph but all the paragraphs until the “Challenges of climate models and infections” section. As said above, the meeting that we are reporting in this article had two goals: (1) making an inventory of opportunities and past research on the topic of climate change and infectious diseases, and (2) identifying key points to research further in the future (which is the last section of the article: “Challenges of climate models and infections”). We have now made this structure clearer in the introduction. Of note though, what you refer to as “background literature” is mostly about the past research conducted in the Asian AAP, in particular.

***Challenges of climate models and infections***

**This section provides a thorough discussion of the challenges from the perspective of infectious disease modelling, but there are major challenges around the uncertainties arising from the boundary conditions that are not discussed (i.e. the climate forcings used for these types of modelling studies). Appropriate expertise within the research team is essential to adequately account for the complexities of climate modelling and its uncertainties. I cannot tell for sure, but the absence of a discussion of the strengths and limitations of climate models implies that this expertise is not represented in the author team. This imbalance should be addressed before finalising any research agenda, in particular before deciding on experimental designs and analytical approaches.**

 Author response: Thank you, this is an important point. Assoc Prof Rachel Lowe from LSHTM and Prof Thanh Ngo-Duc from Hanoi University of Science and Technology were present at the meeting and have expertise in climate models. However, we recognised that this was an under-represented and key area, so we have now expanded our collaboration to climate scientists in the department of Physics at Oxford University and in the European Centre for Medium-Range Weather Forecast (ECMWF) which provides the world’s leading operational global numerical weather prediction on time scales from days, weeks, months, and seasons.

***Future directions and suggested research agenda for the Asian AAPs***

***1) The need for high quality, long term, longitudinal data***

**The authors discuss the need for data at multiple spatial scales and densities. Another major challenge with using longitudinal population data for climate-health research is that the two datasets are often sampled at inappropriate temporal frequencies to capture the relationships between climate/weather drivers and health outcomes. Did the participants discuss these challenges and how to overcome them to achieve their research agenda? They may wish to consult a recent report produced for the Wellcome Trust on this topic.**

 Author response: That is an excellent point that we have indeed identified and discussed extensively after the meeting. In fact, this specific point was listed in our research agenda that we presented to Wellcome in March during their evaluation of our research agenda to begin October 2022. One line of investigation we want to develop in order to address this issue involves the combination of (i) mechanistic models of the effect of climatic variables on the biological processes of these epidemiological systems, and (ii) more agnostic machine-learning-type of modelling in order to identify relevant proxies across scales. The general approach is inspired by methodologies currently developed in downscaling research.

***2) The need for local and international interdisciplinary coloration***

**Has the author team considered including the relevant National Meteoroligcal Services, not just researchers? This would be advisable.**

 Author response: Yes, we have.

***4) Need for individual as well as population level research***

**“In regions experiencing higher ambient temperatures, heat stress causes many health impacts including deranged physiological processes, such as water handling and electrolyte balance.” What is meant by ‘ambient’ in this sentence? Deaths occur from the heat at relatively moderate temperatures, not only extremes.**

 Author response: Ambient temperature is the temperature to which people are exposed to or are directly experiencing. These temperatures could be quite different from temperatures from meteorological stations that are measured in very specific controlled conditions. We have added this explanation to the manuscript.

***5) The need for community engagement***

**The participants identified three goals of community engagement. Supporting operational disease control programs was not mentioned. Did the participants discuss this? Supporting operational disease programming would surely represent an important perspective when setting the research agenda if the intention is to reduce climate-related disease burdens (see also comments below).**

 Author response: Certainly. But this specific aspect has already been one of our key research agendas for many years in the AAP. What we wanted to focus on in the meeting and this article are the aspects that are specifically related to climate change and that are new to our research agenda.

***Box: Next steps and priorities***

**This box is a useful addition to the article but would benefit from being specified more precisely. How will these be achieved? The second point, in particular, is very far-reaching. It seems that you have already prioritised climate-sensitive diseases in this paper, and by the topics that you chose to discuss during the meeting. Is this point more about better understanding and quantifying the risks from these diseases?**

 Author response: Yes, and possibly identifying others that we may have missed in our meeting. Again, the diseases identified in this article are informed by our past research in the programme (which was not climate change oriented). For that reason, it is very possible that we currently have a biased view of the situation. The second point is to fix such a potential issue.

**This is the first place where interventions are mentioned. If the ultimate aim is to reduce climate-related disease risks, surely supporting practical disease programming and operations should be important to the research agenda. It is of course necessary to understand climate-health associations via traditional epidemiological research, but also to understand both where the entry points are for designing new interventions and (often overlooked) how climate could interfere with existing disease control and elimination efforts. Reducing these risks will involve some use of climate data and potentially forecasts, and research questions can be designed with these interventions in mind. Failing to include them at this stage would miss an important opportunity to ensure that the research outcomes are useful for developing effective interventions.**

 Author response: Thank you, and we very much agree with this point. Specific interventions were not discussed at this meeting, but the need to design and test future interventions to reduce climate-related disease was included in the meeting outputs (P12-13). Since the meeting, we have started work on various projects on climate and health interventions, including assessing early action protocols in heat-vulnerable populations in Vietnamese cities. We are also working on facilitating dengue control measures by developing a dengue forecasting tool that will combine probabilistic forecasts of weather and near-term climate conditions with mathematical models of dengue transmission.

**One further question/clarification: why are youth groups specifically the focus of the engagement about climate and mental health?**

 Author response: It is well established that climate anxiety is a prominent issue for children and young people; they stand to suffer the worst consequences but have little formal power to limit the damage. Since the OUCRU public engagement group has built up well-established and fruitful links with young people via schools and online platforms over the past decade, we have identified this as a network upon which engagement work can build. Other age groups will not be excluded from wider engagement projects.
